# Assessment of the severity of diabetic polyneuropathy aids in predicting the risk of developing diabetic complications in patients with untreated diabetes

**DOI:** 10.3389/fendo.2024.1380970

**Published:** 2024-03-15

**Authors:** Shuji Horinouchi, Takahisa Deguchi, Miki Mukai, Ayako Ijuin, Yudai Kawamoto, Yoshihiko Nishio

**Affiliations:** ^1^ Department of Diabetes and Endocrine Medicine, Kagoshima City Hospital, Kagoshima, Japan; ^2^ Department of Diabetes and Endocrine Medicine, Kagoshima University Graduate School of Medical and Dental Sciences, Kagoshima, Japan

**Keywords:** diabetic polyneuropathy, Baba’s classification, nerve conduction study, diabetic complications, untreated diabetes

## Abstract

This study aimed to determine the efficacy of assessing the severity of diabetic polyneuropathy (DPN) in patients with untreated diabetes. Seventy-two patients with untreated type 2 diabetes who were hospitalized for glycemic control were enrolled and divided into the following two groups: patients who had no prior diagnosis and patients who were unattended or had discontinued treatment. Electrophysiological criteria consistent with Baba’s classification were used to diagnose and assess the severity of DPN. The patients were divided into three subgroups: no DPN (stage 0), mild DPN (stage 1), and moderate or more-severe DPN (stages 2–4). Intergroup comparisons were performed for the clinical characteristics and the results of the nerve conduction studies. Twenty-two (30%), 25 (35%), and 25 (35%) patients were categorized into the no DPN, mild DPN, and moderate or more-severe DPN subgroups, respectively. The number of patients who were unattended or had discontinued treatment in the moderate or more-severe DPN subgroup was significantly higher than that in the no DPN subgroup. The patients in the moderate or more-severe DPN subgroup had an increased risk of developing diabetic retinopathy and nephropathy, with odds ratios of 19.5 and 11.0 for advanced stages of retinopathy and nephropathy, respectively. Thus, the assessment of the severity of DPN could aid in the prediction of the risk of developing diabetic complications in patients with untreated diabetes.

## 1 Introduction

The prevalence of type 2 diabetes has been increasing in Japan, with a national survey estimating that approximately 10 million individuals in Japan have diabetes (1). Approximately 23% of patients with diabetes (21.3% of men and 25.9% of women) are untreated (1). Patients with type 2 diabetes often experience few subjective symptoms; consequently, diabetes often remains undetected, and diabetic complications tend to progress without being detected (2, 3). A review of the clinical background of patients with advanced diabetic retinopathy at presentation revealed that 107 individuals, including 65 patients with untreated diabetes (22 were unattended, 20 had discontinued treatment, and 23 had no prior diagnosis) (4), had pre-proliferative or proliferative diabetic retinopathy.

Diabetic polyneuropathy (DPN) is the most prevalent chronic complication of diabetes that occurs during the earliest stages of the disease (5, 6). The early stages of DPN are associated with few subjective symptoms, with generally only mild numbness or abnormal sensations below the knee and in both legs; however, as DPN progresses, it becomes a serious complication that causes neuropathic pain, autonomic neuropathy, diabetic foot, and sudden death. Diabetic bladder dysfunction caused by autonomic neuropathy, occurs with relatively frequency. This can present as incomplete bladder emptying, an increased postvoid residual, decreased peak urinary flow rate, bladder overdistention, and urine retention, which can lead to urinary tract infections (7, 8). Severe DPN has a significant impact on the quality of life and prognosis of patients with diabetes. Baba et al. proposed the use of a combination of sural sensory nerve action potential (SNAP) amplitude, tibial compound muscle action potential (CMAP) amplitude, and velocity system indices [such as tibial F-wave latency, tibial motor nerve conduction velocity (MCV), and sural sensory nerve conduction velocity (SCV) in the lower limb] for the diagnosis of the severity of DPN (9). The severity of DPN diagnosed according to this classification was associated with the prognosis of patients with diabetes in a recent 5-year prospective study (10).

The association between diabetic retinopathy and diabetic neuropathy is correlated with the severity of DPN and alterations in retinal thickness (11). Thus, evaluating the severity of DPN at the initial visit may be essential in all diabetic patients. Evaluating the severity of DPN may be especially useful in predicting the prognosis of patients with untreated diabetes at high risk of developing diabetic complications. Therefore, this study aimed to investigate the severity of DPN in patients with untreated diabetes and examine the association between the development of diabetic complications and the severity of DPN.

## 2 Materials and methods

### 2.1 Patients

This study was conducted at the Kagoshima City Hospital, Kagoshima, Japan, between June 2015 and May 2021. Among the 3370 patients with type 2 diabetes who visited our department for the first time, 649 patients were untreated. Among these 649 patients, 117 were hospitalized for the management of hyperglycemia during the study period, and 72 (28 women and 44 men) participated in the current study. Forty-five patients, comprising 38 patients who did not undergo nerve conduction studies (NCSs), six patients aged >75 years, and one patient with a neuromuscular disease, were excluded from the study. All patients underwent routine biochemical and hematological tests and screening for diabetic complications. In addition, drug treatment was also analyzed after hospitalization. The untreated patients were divided into two groups: patients with no prior diagnosis and patients who were unattended or had discontinued treatment. Patients classified as having no prior diagnosis comprised those who i) had not previously been diagnosed with diabetes following any physical examination or hospital visit and ii) those who had not undergone a physical examination and were diagnosed with diabetes for the first time on visiting our department. Unattended patients were defined as patients diagnosed with diabetes at a medical checkup who did not initiate treatment. Discontinuation of treatment was defined as a failure to visit the hospital for ≥1 year after commencing treatment.

### 2.2 Nerve conduction study

All patients underwent conventional sensory and motor NCSs. The median, ulnar, tibial, and sural nerves of the upper and lower extremities were examined. NCSs were performed using a standard electromyographic device with stimulating and recording electrodes (Viking Select; Nicolet Biomedical Japan, Tokyo, Japan). The skin temperature was maintained above 32°C and at 31°C on the forearm and mid-leg, respectively. The American Academy of Electrodiagnostic Medicine defines DPN as a distal symmetric sensorimotor polyneuropathy. Patients with abnormalities in any attribute of nerve conduction in two separate nerves, one of which must be the sural nerve, were diagnosed with DPN (12). The classification proposed by Baba was also used as a diagnostic reference in addition to the authorized diagnostic criterion. DPN was classified into five stages based on the severity classification of DPN in this classification system: stage 0, normal with no NCS abnormalities; stage 1, mild neuropathy with tibial MCV of <42 m/s, sural SCV of <42 m/s, tibial minimal F-wave latency (> [12.8 + 0.22 × height (cm)] ms), or the presence of an A wave; stage 2, moderate neuropathy with a sural SNAP amplitude of <5 µV; stage 3, moderate-to-severe neuropathy with a sural SNAP amplitude of <5 µV and a tibial CMAP amplitude ranging between ≥2 and <5 mV; and stage 4, severe neuropathy with a sural SNAP amplitude of <5 µV and a tibial CMAP amplitude of <2 mV (13). The patients were further divided into the following three subgroups according to these grades: no DPN (stage 0), mild DPN (stage 1), and moderate or more-severe DPN (stages 2–4). Intergroup comparisons were performed for the clinical characteristics of the patients and the NCS results.

### 2.3 Statistical analysis

Data are presented as the mean and standard deviation. Excel 2021 (Microsoft, Redmond, WA, USA) with the add-in software Statcel 3 (OMS, Tokyo, Japan) and EZR (Saitama Medical Center, Jichi Medical University, Saitama, Japan) were used to perform all statistical analyses (14). Significant differences among the three groups were compared using one-way analysis of variance and the Tukey–Kramer or Steel–Dwass post-hoc test. The adjusted odds ratios with the 95% confidence intervals (CIs) were calculated using the logistic regression model to determine the risk of developing diabetic retinopathy and diabetic nephropathy. A P-value of <0.05 was considered statistically significant.

## 3 Results

### 3.1 Clinical characteristics of the patients

**Table 1** presents the demographic data of the 72 participants. The mean age of the participants was 54.3 ± 11.4 years. The mean duration of diabetes was 7.2 ± 6.6 years. Thirty-one of the 72 participants had no prior diagnosis of diabetes. The remaining 41 were unattended or had discontinued treatment. The mean glycosylated hemoglobin (HbA1c) level was 11.1% ± 2.0%. Retinopathy, nephropathy, and macroangiopathy were detected in 30 (42%), 33 (46%), and eight (11%) patients, respectively. Three (4%) patients had diabetic foot. Twenty-two (30%), 25 (35%), 18 (25%), three (4%), and four (6%) patients were categorized as having no DPN (stage 0), mild DPN (stage 1), moderate DPN (stage 2), moderate to severe DPN (stage 3), and severe DPN (stage 4), respectively. The proportion of males among patients who were unattended or had discontinued treatment was significantly higher. The participants who were unattended or had discontinued had more advanced stages of diabetic retinopathy, diabetic nephropathy, or DPN than the participants with no prior diagnosis (all P < 0.01).

**Table 1:** Clinical characteristics of the patients.

	Total patients	No prior diagnosis	Unattended or interrupted treatment
n	72	31	41
Male sex (%)	61.1	45.2	73.2**
Age (years)	54.3 ± 11.4	54.8 ± 11.9	53.8 ± 11.1
BMI (kg/m^2^)	26.4 ± 6.0	27.4 ± 7.0	25.7 ± 5.2
Diabetes duration (years) (n = 40)	7.2 ± 6.6 (n = 40)	–	7.2 ± 6.6 (n = 40)
Untreated duration (years) (n = 40)	5.1 ± 5.7 (n = 40)	–	5.1 ± 5.7 (n = 40)
HbA1c (%)	11.1 ± 2.0	10.6 ± 2.3	11.5 ± 1.6
Urine CPR (µg/day)	66.5 ± 43.4	77.1 ± 40.9	58.4 ± 44.1
eGFR (mL/min/1.73 m^2^)	94.1 ± 31.2	93.6 ± 32.3	94.4 ± 30.7
Non-insulin/insulin (n)	50/22	25/6	25/16
Diabetic retinopathy; NDR/SDR/PPDR/PDR (n)	42/8/9/13	26/2/1/2	16/6/8/11**
Diabetic nephropathy; 1/2/3/4/5^†^ (n)	39/22/9/2/0	22/7/2/0	17/15/7/2**
Diabetic polyneuropathy; 0/1/2/3/4^‡^ (n)	22/25/18/3/4	13/13/4/0/1	9/12/14/3/3**
Macroangiopathy (%)	11.1	9.7	12.2
Diabetic foot (%)	4.2	0	7.3

BMI, body mass index; HbA1c glycosylated hemoglobin, CPR, C-peptide immunoreactivity; eGFR, estimated glomerular filtration rate; NDR, non-diabetic retinopathy; SDR, simple diabetic retinopathy; PPDR, preproliferative diabetic retinopathy; PDR, proliferative diabetic retinopathy.

^†^Diagnosed and categorized using the Classification of Diabetic Nephropathy 2014 established by the Joint Committee on Diabetic Nephropathy (15).

^‡^Diagnosed and categorized using the classification proposed by Baba for the severity of DPN.

^**^P < 0.01 versus the no prior diagnosis group.

### 3.2 Clinical characteristics of the patients stratified according to the classification proposed by Baba

**Table 2** presents the clinical characteristics of the 72 patients stratified according to the classification proposed by Baba (9, 13). The diagnosis was consistent with the authorized diagnostic criteria and the classification proposed by Baba in all cases. No significant differences were observed among the three subgroups in terms of the estimated glomerular filtration rate or the prevalence of macroangiopathy or diabetic foot. The body mass index and lower urinary C-peptide immunoreactivity of the patients with moderate or more-severe DPN were significantly lower than those of the patients without DPN (both P < 0.01). The HbA1c values of the patients with moderate or more-severe DPN were significantly higher than those in the no DPN subgroup (P < 0.05). The proportion of patients who were unattended or had interrupted treatment in the moderate or more-severe-DPN group was significantly higher than that in the no-DPN group (P < 0.05). The use of insulin was similar among all three subgroups. The stages of diabetic retinopathy and diabetic nephropathy in the moderate or more-severe-DPN subgroup were significantly more advanced than those in the no-DPN and mild-DPN subgroups (retinopathy: both P < 0.01, nephropathy: P < 0.01 for no DPN and P < 0.05 for mild DPN). Other than age and HbA1c values, no significant differences were observed between the no DPN and mild-DPN subgroups in terms of clinical presentation.

**Table 2:** Clinical characteristics of the patients stratified according to the classification proposed by Baba.

	Severity of DPN		
	No DPN	Mild DPN	Moderate or more-severe DPN
	(stage 0)	(stage 1)	(stages 2–4)
n	22	25	25
Male sex (%)	68.2	40.0	76.0^a^
Age (years)	49.6 ± 12.7	58.1 ± 9.8^*^	54.6 ± 10.6
BMI (kg/m^2^)	29.8 ± 5.4	26.0 ± 6.1	23.9 ± 5.3^**^
No prior diagnosis/unattended or interrupted treatment (n)	13/9	13/12	5/20^*^
Diabetes duration (years) (n = 40)	3.7 ± 3.4 (n = 9)	6.6 ± 8.1 (n = 12)	9.2 ± 6.1 (n = 19)
Untreated duration (years) (n = 40)	2.2 ± 1.5 (n = 9)	4.9 ± 8.0 (n = 12)	6.5 ± 4.9 (n = 19)
HbA1c (%)	10.1 ± 1.8	11.7 ± 2.2^*^	11.5 ± 1.6^*^
Urine CPR (µg/day)	82.8 ± 49.7	71.7 ± 40.2	45.6 ± 32.1^**^
eGFR (mL/min/1.73 m^2^)	99.2 ± 28.3	93.6 ± 23.9	90.0 ± 39.5
Non-insulin/insulin (n)	18/4	17/8	15/10
Diabetic retinopathy; NDR/SDR/PPDR/PDR (n)	20/1/0/1	16/6/1/2	6/1/8/10^**b^
Diabetic nephropathy; stage 1/2/3/4^†^ (n)	16/5/1/0	15/9/0/1	8/8/8/1^**a^
Macroangiopathy (%)	4.5	24.0	4.0
Diabetic foot (%)	0	0	12.0

Data are expressed as the mean ± standard deviation, n, or %.

DPN, diabetic polyneuropathy; BMI, body mass index; HbA1c, glycosylated hemoglobin; CPR, C-peptide immunoreactivity; eGFR, estimated glomerular filtration rate; NDR, non-diabetic retinopathy; SDR, simple diabetic retinopathy; PPDR, preproliferative diabetic retinopathy; PDR, proliferative diabetic retinopathy.

^†^Diagnosed and categorized using the Classification of Diabetic Nephropathy 2014 established by the Joint Committee on Diabetic Nephropathy (15).

^*^P < 0.05, ^**^P < 0.01 versus the no-DPN group; ^a^P < 0.05, ^b^P < 0.01 versus the mild-DPN group.

### 3.3 NCS parameters

**Table 3** presents the results of the lower limb NCS for the 72 participants. The tibial MCV and sural SCV of the moderate or more-severe-DPN subgroup were significantly lower than those of the no-DPN and mild-DPN subgroups (P < 0.01). The tibial F-wave latency of the moderate or more-severe-DPN subgroup was significantly longer than those of the no-DPN and mild-DPN subgroups (P < 0.01). The amplitudes of the tibial CMAP and sural SNAP in the moderate or more severe-DPN subgroup were significantly smaller than those in the no-DPN and mild-DPN groups (CMAP: P < 0.01 for no DPN and P < 0.05 for mild DPN, SNAP: both P < 0.01). The tibial MCV was significantly lower, the tibial F-wave latency was significantly longer, and the sural SNAP was smaller in the mild-DPN group than those in the no-DPN group (all P < 0.01).

**Table 3:** Comparison of the results of the lower limb nerve conduction studies in patients stratified according to the classification proposed by Baba.

	Severity of DPN		
	No DPN	Mild DPN	Moderate or more-severe DPN
	(stage 0)	(stage 1)	(stages 2–4)
n	22	25	25
Tibial nerve			
CMAP (mV)	14.9 ± 4.7	11.6 ± 4.4	7.8 ± 5.4^**a^
MCV (m/s)	46.2 ± 3.0	42.3 ± 1.9^**^	37.2 ± 4.2^**b^
F-wave latency (ms)	46.2 ± 2.2	50.7 ± 3.5^**^	56.1 ± 6.6^**b^
Sural nerve			
SNAP (µV)	16.4 ± 6.8	11.0 ± 4.6^**^	2.7 ± 1.7^**b^
SCV (m/s)	49.8 ± 3.9	47.1 ± 4.5	34.9 ± 16.2^**b^

Data are expressed as the mean ± standard deviation or n.

DPN, diabetic polyneuropathy; CMAP, compound muscle action potential; MCV, motor nerve conduction velocity; SNAP, sensory nerve action potential; SCV, sensory nerve conduction velocity.

^*^P < 0.05, ^**^P < 0.01 versus the no-DPN group; ^a^P < 0.05, ^b^P < 0.01 versus the mild-DPN group.

### 3.4 Risk of developing retinopathy or nephropathy in multivariate logistic regression analysis

The risk of developing diabetic retinopathy and diabetic nephropathy among patients who were unattended or had discontinued treatment was calculated as odds ratios after adjusting for the potential confounder of diabetes duration (**Table 4**). The risk of developing diabetic retinopathy was higher in the moderate or more-severe-DPN subgroup, with an adjusted odds ratio of 7.56 (95% CI: 1.590–36.0, P = 0.011). The moderate or more-severe DPN subgroup also showed significant associations with the risk of progression of retinopathy and nephropathy, with adjusted odds ratios of 19.5 (95% CI: 3.660–104.0, P < 0.001) and 11.0 (95% CI: 1.150–106.0, P = 0.037) for retinopathy and nephropathy, respectively. The duration of diabetes showed no associations with the risk of developing diabetic retinopathy or diabetic nephropathy.

**Table 4:** Risk of developing retinopathy or nephropathy in the multivariate logistic regression analysis.

Factor	Multivariate analysis		
	Odds ratio	95% CI	P
Retinopathy			
Diabetes duration	1.040	0.926–1.170	0.493
Moderate or more-severe DPN	7.560	1.590–36.000	0.011
Stages 2–4			
Retinopathy; PPDR or PDR			
Diabetes duration	1.060	0.941–1.200	0.326
Moderate or more-severe DPN	19.500	3.660–104.000	<0.001
Stages 2–4			
Nephropathy			
Diabetes duration	1.090	0.953–1.240	0.210
Moderate or more-severe DPN	1.130	0.281–4.540	0.863
Stages 2–4			
Nephropathy; stage 3 or 4			
Diabetes duration	1.020	0.887–1.160	0.819
Moderate or more-severe DPN	11.000	1.150–106.000	0.037
Stages 2–4			

CI, confidence interval; DPN, diabetic polyneuropathy; PPDR, preproliferative diabetic retinopathy; PDR, proliferative diabetic retinopathy.

## 4 Discussion

To the best of our knowledge, this study is the first to investigate the validity of assessing the severity of DPN for predicting the risk of developing diabetic complications in patients with untreated diabetes. The present study revealed that the risk of developing diabetic complications was related to the DPN severity classification system proposed by Baba. Patients with moderate or more-severe DPN had a higher risk of developing advanced stages of diabetic retinopathy and diabetic nephropathy.

The Michigan Neuropathy Screening Instrument (16) and the Toronto Consensus (17) have been used for the diagnosis of DPN worldwide. The use of the simplified diagnostic criteria of the Japanese Study Group on Diabetic Neuropathy (18) has been recommended in routine practice in Japan. DPN was detected in 35.8% of patients with type 2 diabetes in Japan according to these criteria in a previous study (19). However, these criteria comprise the physical signs and symptoms of peripheral neuropathy and cannot be used to determine the severity of DPN.

NCSs provide the most objective and quantitative method for diagnosing DPN (20). DPN was detected in 70% of patients with poorly controlled untreated diabetes via NCSs in the present study. The prevalence of mild DPN was 35%. The prevalence of moderate or more-severe DPN was also 35%. The number of patients with stage 3 and 4 disease was small; however, the frequency of severe DPN among the participants was comparable with that reported in other Japanese studies (21, 22). This finding indicates the high prevalence of DPN among patients with untreated diabetes and suggests that NCS shows excellent sensitivity for the detection of DPN. However, NCSs require expensive equipment and an advanced examination technique. Moreover, they are only available in limited facilities at present. Consequently, a point-of-care device, DPNCheck™ (NeuroMetrix Inc., Waltham, MA, USA), was developed to test only the sural nerve to overcome the lack of versatility of NCSs (23, 24). The results of DPNCheck^™^ are highly reproducible and correlate well with a standard electromyographic system (25, 26). Kamiya et al. developed a multiple regression model to predict the severity of DPN based on the classification system proposed by Baba that used sural nerve conduction data in DPNCheck^™^ (21). The model could effectively diagnose moderate or more-severe DPN (stages 2–4) (21). The use of this model may facilitate the assessment of the severity of DPN in facilities where NCSs cannot be performed.

The prevalence of severe diabetic complications has been increasing among patients who do not visit a physician owing to social and economic reasons. A lack of understanding of treatment priorities, lack of awareness regarding the disease, and financial burdens have been identified as reasons for discontinuing treatment (27). Severe complications from proliferative diabetic retinopathy, such as tractional retinal detachment, tend to develop in patients who discontinue treatment (28). More effective medical care and guidance can be provided by identifying untreated patients at a high risk of diabetic complications at the initial visit. Assessing the severity of DPN aids in predicting the risk of developing diabetic complications and preventing the progression of complications and the development of diabetic foot. However, owing to the difficulty in implementing NCSs, their use for the prediction of retinopathy or nephropathy is not feasible. Thus, objective assessment of the severity of DPN using DPNCheck^™^ in various medical settings could aid in the detection of high-risk patients and the prevention of severe diabetes in the future.

This study is limited by its single-center design and relatively small sample size. The number of patients with stage 3 and 4 disease was insufficient to facilitate statistical analyses; consequently, differences among stages 0, 1, and 2–4 were analyzed. In addition, the analysis could not distinguish moderate DPN from moderate-to-severe or severe DPN. Participants with moderate-to-severe or severe DPN may be at increased risk of developing diabetic complications, including diabetic foot. Therefore, the generalizability of these findings may be limited, and additional data must be accumulated on participants with advanced DPN. Further studies with large sample sizes are required to determine the relevance of the findings of the present study more accurately.

In conclusion, assessment of the severity of DPN aids in the prediction of the risk of developing diabetic complications in patients with untreated diabetes. These findings could be used as guidelines to identify patients with untreated diabetes at high risk of developing diabetic complications at the initial visit to the hospital.

## Data availability statement

The raw data supporting the conclusions of this article will be made available by the authors, without undue reservation.

## Ethics statement

The studies involving humans were approved by the Kagoshima City Hospital Ethics Committee. The studies were conducted in accordance with the local legislation and institutional requirements. The participants provided their written informed consent to participate in this study.

## Author contributions

SH: Writing – review & editing, Writing – original draft. TD: Writing – review & editing. MM: Writing – review & editing. AI: Writing – review & editing. YK: Writing – review & editing. YN: Writing – review & editing.

## References

[1] Ministry of Health. Labour and Welfare (Japan) (2016). Available online at: https://www.mhlw.go.jp/bunya/kenkou/eiyou/h28-houkoku.html (Accessed August 8, 2018).

[2] Harris MI Klein R Welborn TA Knuiman MW. Onset of NIDDM occurs at least 4-7 yr before clinical diagnosis. Diabetes Care. (1992) 15:815–9. doi: 10.2337/diacare.15.7.815 1516497

[3] Harris MI. Undiagnosed NIDDM: clinical and public health issues. Diabetes Care. (1993) 16:642–52. doi: 10.2337/diacare.16.4.642 8462395

[4] Gemma R Kitahara K Kono E Imoto M Ikeya A Iwaki H. Clinical background factors of patients with advanced diabetic retinopathy at the first presentation. J Japan Diabetes Soc. (2015) 58:192–7. doi: 10.11213/tonyobyo.58.192

[5] Dyck PJ Herrmann DN Staff NP Dyck PJ. Assessing decreased sensation and increased sensory phenomena in diabetic polyneuropathies. Diabetes. (2013) 62:3677–86. doi: 10.2337/db13-0352 PMC3806590 24158999

[6] Pop-Busui R Boulton AJ Feldman EL Bril V Freeman R Malik RA. Diabetic neuropathy: a position statement by the American Diabetes Association. Diabetes Care. (2017) 40:136–54. doi: 10.2337/dc16-2042 PMC6977405 27999003

[7] Vinik AI Maser RE Mitchell BD Freeman R. Diabetic autonomic neuropathy. Diabetes Care. (2003) 26:1553–79. doi: 10.2337/diacare.26.5.1553 12716821

[8] Micle O Antal L Naghi P Tica O Zaha DC Zdrîncă MM. The prevalence of urinary tract infections in pregnancy and implications on foetal development. Farmacia. (2020) 68:463–9. doi: 10.31925/farmacia.2020.3.11

[9] Baba M Suzuki C. Electrophysiological grading of diabetic polyneuropathy by nerve conduction study. J Clin Exp Med. (2013) 41:143–50. doi: 10.11422/jscn.41.143

[10] Baba M Suzuki C Ogawa Y. Severity grading system of diabetic neuropathy in type-2 diabetes by nerve conduction study: five-year prospective study on occurrence of diabetic foot, macroangiopathic events, and eventual death. Jpn J Clin Neurophysiol. (2018) 46:71–7. doi: 10.11422/jscn.46.71

[11] Yamada Y Himeno T Tsuboi K Shibata Y Kawai M Asada-Yamada Y. Alterations of retinal thickness measured by optical coherence tomography correlate with neurophysiological measures in diabetic polyneuropathy. J Diabetes Investig. (2021) 12:1430–41. doi: 10.1111/jdi.13476 PMC8354512 33300294

[12] England JD Gronseth GS Franklin G Miller RG Asbury AK Carter GT. Distal symmetric polyneuropathy: a definition for clinical research: report of the American Academy of Neurology, the American Association of Electrodiagnostic Medicine, and the American Academy of Physical Medicine and Rehabilitation. Neurology. (2005) 64:199–207. doi: 10.1212/01.WNL.0000149522.32823.EA 15668414

[13] Himeno T Kamiya H Nakamura J. Lumos for the long trail: strategies for clinical diagnosis and severity staging for diabetic polyneuropathy and future directions. J Diabetes Investig. (2020) 11:5–16. doi: 10.1111/jdi.13173 PMC6944828 31677343

[14] Kanda Y. Investigation of the freely available easy-to-use software ‘EZR’ for medical statistics. Bone Marrow Transplant. (2013) 48:452–8. doi: 10.1038/bmt.2012.244 PMC3590441 23208313

[15] Haneda M Utsunomiya K Koya D Babazono T Moriya T Makino H. A new Classification of Diabetic Nephropathy 2014: a report from Joint Committee on Diabetic Nephropathy. J Diabetes Investig. (2015) 6:242–6. doi: 10.1111/jdi.12319 PMC4364860 25802733

[16] Feldman EL Stevens MU Thomas PK Brown MB Canal N Greene DA. A practical two-step quantitative clinical and electrophysiological assessment for the diagnosis and staging of diabetic neuropathy. Diabetes Care. (1994) 17:1281–9. doi: 10.2337/diacare.17.11.1281 7821168

[17] Tesfaye S Boulton AJ Dyck PJ Freeman R Horowitz M Kempler P. Diabetic neuropathies: update on definitions, diagnostic criteria, estimation of severity, and treatments. Diabetes Care. (2010) 33:2285–93. doi: 10.2337/dc10-1303 PMC2945176 20876709

[18] Yasuda H Sanada M Kitada K Terashima T Kim H Sakaue Y. Rationale and usefulness of newly devised abbreviated diagnostic criteria and staging for diabetic polyneuropathy. Diabetes Res Clin Pract. (2007) 77:S178–83. doi: 10.1016/j.diabres.2007.01.053 17478005

[19] Satoh J Baba M Yagihashi S Suda T Tominaga M Daimon M. Frequency of diabetic polyneuropathy (DPN) and clinical significance of Achilles tendon reflex in diagnosis of DPN -survey of 15,000 patients in Tohoku, Japan. J Japan Diabetes Soc. (2007) 50:799–806. doi: 10.11213/tonyobyo.50.799

[20] Kohara N Kimura J Kaji R Goto Y Ishii J Takiguchi M. F-wave latency serves as the most reproducible measure in nerve conduction studies of diabetic polyneuropathy: multicentre analysis in healthy subjects and patients with diabetic polyneuropathy. Diabetologia. (2000) 43:915–21. doi: 10.1007/s001250051469 10952465

[21] Kamiya H Shibata Y Himeno T Tani H Nakayama T Murotani K. Point-of-care nerve conduction device predicts the severity of diabetic polyneuropathy: A quantitative, but easy-to-use, prediction model. J Diabetes Investig. (2021) 12:583–91. doi: 10.1111/jdi.13386 PMC8015817 32799422

[22] Mikura K Kodama E Iida T Imai H Hashizume M Kigawa Y. Association between sarcopenia and the severity of diabetic polyneuropathy assessed by nerve conduction studies in Japanese patients with type 2 diabetes mellitus. J Diabetes Investig. (2022) 13:1357–65. doi: 10.1111/jdi.13788 PMC9340862 35271762

[23] Perkins BA Grewal J Ng E Ngo M Bril V. Validation of a novel point-of-care nerve conduction device for the detection of diabetic sensorimotor polyneuropathy. Diabetes Care. (2006) 29:2023–7. doi: 10.2337/dc08-0500 16936147

[24] Lee JA Halpern EM Lovblom LE Yeung E Bril V Perkins BA. Reliability and validity of a point-of-care sural nerve conduction device for identification of diabetic neuropathy. PloS One. (2014) 9:e86515. doi: 10.1371/journal.pone.0086515 24466129 PMC3899274

[25] Hirayasu K Sasaki H Kishimoto S Kurisu S Noda K Ogawa K. Difference in normal limit values of nerve conduction parameters between Westerners and Japanese people might need to be considered when diagnosing diabetic polyneuropathy using a point-of-care sural nerve conduction device (NC-stat^®^/DPNCheck™). J Diabetes Investig. (2018) 9:1173–81. doi: 10.1111/jdi.12818 PMC6123044 29430866

[26] Shibata Y Himeno T Kamiya T Tani H Nakayama T Kojima C. Validity and reliability of a point-of-care nerve conduction device in diabetes patients. J Diabetes Investig. (2019) 10:1291–8. doi: 10.1111/jdi.13007 PMC6717804 30659760

[27] Japanese Practice Guidance to Improve Patients’ Adherence to Appointments for Diabetes Care (2019). Available online at: https://human-data.or.jp/wp/wp-content/uploads/2018/07/dm_jushinchudan_guide43_e.pdf (Accessed July 15, 2019).

[28] Obeid A Su D Patel SN Uhr JH Borkar D Gao X. Outcomes of eyes lost to follow-up with proliferative diabetic retinopathy that received panretinal photocoagulation versus intravitreal anti-vascular endothelial growth factor. Ophthalmology. (2019) 126:407–13. doi: 10.1016/j.ophtha.2018.07.027 30077614

